# Context-dependent coloration of prey and predator decision making in contrasting light environments

**DOI:** 10.1093/beheco/arab111

**Published:** 2021-10-18

**Authors:** Ossi Nokelainen, Francisko de Moraes Rezende, Janne K Valkonen, Johanna Mappes

**Affiliations:** 1 Department of Biological and Environmental Science, University of Jyväskylä, Jyväskylä, Finland; 2 Organismal and Evolutionary Biology Research Program, Faculty of Biological and Environmental Sciences, University of Helsinki, Viikki Biocenter 3, Helsinki, Finland

**Keywords:** behavior, cognition, color vision, psychology, receptor-noise-limited model, signal

## Abstract

A big question in behavioral ecology is what drives diversity of color signals. One possible explanation is that environmental conditions, such as light environment, may alter visual signaling of prey, which could affect predator decision-making. Here, we tested the context-dependent predator selection on prey coloration. In the first experiment, we tested detectability of artificial visual stimuli to blue tits (*Cyanistes caeruleus*) by manipulating stimulus luminance and chromatic context of the background. We expected the presence of the chromatic context to facilitate faster target detection. As expected, blue tits found targets on chromatic yellow background faster than on achromatic grey background whereas in the latter, targets were found with smaller contrast differences to the background. In the second experiment, we tested the effect of two light environments on the survival of aposematic, color polymorphic wood tiger moth (*Arctia plantaginis*). As luminance contrast should be more detectable than chromatic contrast in low light intensities, we expected birds, if they find the moths aversive, to avoid the white morph which is more conspicuous than the yellow morph in low light (and vice versa in bright light). Alternatively, birds may attack first moths that are more detectable. We found birds to attack yellow moths first in low light conditions, whereas white moths were attacked first more frequently in bright light conditions. Our results show that light environments affect predator foraging decisions, which may facilitate context-dependent selection on visual signals and diversity of prey phenotypes in the wild.

## Teaser

Light environments are constantly changing and may alter visual appearance of prey, but also bias predators’ decision making. Our findings using blue tits in visual search tasks and the wood tiger moth prey under two light environments demonstrate that birds show context-dependent predatory behavior. This suggests that light environments can play a major selective role and influence visual signaling in the wild.

## INTRODUCTION

Visual signaling can powerfully shape the outcomes of sexual, social, and natural selection (Maynard Smith and Harper 2003; Stevens 2013; Cronin et al. 2014). For example, the interactions amongst color and light environment led to cichlid fish island populations diversification through sensory drive (Seehausen et al. 2008). In birds, intraspecific variation in plumage coloration is linked to light environment use (McNaught 2002; Price 2010; Tate et al. 2016). Broadly speaking, as downwelling irradiance gets altered by vegetation (e.g., as illustrated by a mosaic of light environments inside forests), it influences those wavelengths that contrast against the respective visual background in that signaling environment (Endler 1993). Elusively, changes in any phase of the sensory experience (i.e., viewing conditions, visual cues, perception, and cognitive processing) may alter how visual cues carry over, favor different spectral properties (i.e., color, saturation, or brightness) in signaling, and facilitate the evolution of prey phenotypes (Endler 1992; Endler and Mappes 2017; Price 2017).

From a standpoint of predator-prey interactions, differences in the reflectance of prey, its luminance and chromatic contrast to the background facilitate the use of visual cues by predators (Roper and Redston 1987; Siddiqi et al. 2004; Prudic et al. 2007; Zylinski and Osorio 2013; White et al. 2015). Conspicuousness and distinctiveness of warning signals (such as color) help predators to form negative associations with secondary defenses (such as foul-tasting chemicals), to remember these associations, and accelerate subsequent decision making when encountering similar prey types (Roper and Redston 1987; Speed 2000; Prudic et al. 2007). For example, Roper and Redston (1987) presented domestic chicks (*Gallus gallus*) with unpalatable red or white beads against red or white backgrounds; conspicuous color combinations led to faster avoidance learning and longer retention of such avoidance than inconspicuous combinations. Also, Prudic et al. (2007) found similar results while offering milkweed bugs (*Oncopeltus fasciatus*) that had their luminance contrast manipulated to Chinese praying mantids (*Tenodera aridifolia sinensis*).

The use of visual information is, however, dependent on predator perception and viewing conditions. Perceptual capability includes eye anatomical properties, photoreceptor sensitivities to different wavelengths, and photoreceptor relative frequencies (Endler 1992; Kelber et al. 2003; Cronin et al. 2014; White and Kemp 2015; Kelber 2019). Most humans are trichromatic and see colors that are approximately in the 400–700 nm range whereas many birds are tetrachromatic and see colors also in the ultraviolet (320–400 nm) range (Cronin and Bok 2016). Some animals are di- or monochromats (or possess more receptor types), which influences how effectively chromatic and luminance information can be processed (Chittka 1996; Troscianko et al. 2017). Importantly, not only the wavelengths matter but also how receivers filter visual information (i.e., receiver psychology) through higher-level cognitive processing and react to it (Rowe 2013; Hutton et al. 2015; Endler and Mappes 2017). For instance, aposematic prey might experience a momentary increase in predation risk due to the conspicuousness of their warning signals before predators learn to avoid them (Lindström et al. 1999; Ruxton et al. 2019). Another important aspect is the environment where the signal is presented. Rojas et al. (2014) showed that signal detectability of the dyeing poison frog (*Dendrobates tinctorius*) depends on the light conditions under which signaling takes place. Thus, a range of conditions influence the attack risk of prey and further, sometimes predators may not act as predicted when their perception is evaluated through vision models.

We investigated how context-dependent visual cues influence predator search behavior and decision-making to attack defended prey in contrasting light environments. We used the Wood tiger moth (*Arctia plantaginis*, Erebidae: Arctiinae) as our model prey. This species has distinctive wing color morphs that its bird predators can detect (Henze et al. 2018), it produces defensive chemicals eliciting predator avoidance, which justifies its status as an aposematic organism (Nokelainen et al. 2012; Hegna et al. 2013; Burdfield-Steel et al. 2018; Rönkä et al. 2018a). We focused on males, which are polymorphic regarding their hind-wing coloration: their hind wings may be yellow (chroma-rich) or white (luminance-rich). We chose Blue tits (*Cyanistes caeruleus*) as predators because their visual physiology is well-understood (Hart et al. 2000), they occur in the same areas as wood tiger moths, and are known to separate wood tiger moths*’* white and yellow color morphs (Rönkä et al. 2018b). It has also been shown that bird communities that inhabit different light environments select for different wood tiger moth warning signals (yellow and white) in nature (Nokelainen et al. 2014).

To test a context-dependent predation hypothesis, we conducted two experiments. First, we tested the context-dependent detectability of artificial visual stimuli by manipulating target luminance contrasts in two visual contexts (chromatic yellow background vs. achromatic grey background) in behavioral assays with blue tits. Then, we measured the irradiance profiles of common boreal forest light environments in the wild and assessed the significance of two contrasting light environments (high vs. low intensity) on the survival of real prey, the polymorphic wood tiger moth, in a simultaneous choice test.

In the first experiment, detectability of artificial visual stimuli in a fixed light environment under luminance and chromatic contexts, we hypothesized that the presence of chromatic context in the visual search task should lead to faster prey detection (Troscianko et al. 2017). Depending on the task difficulty, the detection response of the birds towards the visual stimuli may show either a linear or non-linear (or categorical) relationship (Caves et al. 2018; Kelber 2019). In the second experiment, we tested the influence of contrasting light environments on the survival of real polymorphic prey. We reasoned that the spectrum of ambient light is biased towards longer wavelengths with high intensity in exposed areas (i.e., forest borders and clearings). Under canopy, on the other hand, lower light intensity and a flattened distribution of ambient light across the spectrum is the norm (Endler 1993). In low light intensities luminance contrast should be more detectable (and salient) than chromatic contrast (Arenas et al. 2014; Cronin et al. 2014; Kelber 2019). Thus, provided that birds have completed avoidance learning on conspicuous, defended prey (Rönkä et al. 2018b), we expected shady conditions to hamper yellow warning signal efficacy (i.e., to get more attacks); in higher-light intensities, on the other hand, chromatic warning signals should work better (i.e., and to get less attacks). Finally, we expected the opposite pattern (i.e., yellow signals being attacked more often in shady conditions, and the opposite in high light intensity) if attacks are more dependent on sheer prey detectability.

## METHODS

### Bird capture and aviary facilities

The following behavioral assays using wild birds (to which we offered artificial stimuli and real prey, *A. plantaginis*) were conducted at the Konnevesi field station (62°36’59.3”N, 26°20’44.2”E). Wild birds (blue tits, *C. caeruleus*) were used with permission from the local authorities. Birds were captured and housed with permission of Central Finland Centre for Economic Development, Transport and Environment (VARELY/294/2015) and a license from the National Animal Experiment Board (ESAVI/9114/04.10.07/2014).

The birds were captured during the winter of 2017–2018. The birds were caught using baited traps, ringed, and kept in indoor aviaries located at the field station under a 12:12h light/dark cycle (for logistic reasons) with access to a varied mix of seeds and water. Upon capture, birds were weighed, sexed, and had their molting pattern analyzed to determine if they were one-year-old (i.e., juveniles) or older (i.e., adults). In the first experiment, we used 15 blue tits. Out of these, two were adult males, one was an adult female, five were juvenile males and seven were juvenile females. In the second experiment, we used 49 blue tits. Out of these, 10 were adult males, 10 were adult females, 16 were juvenile males and 13 were juvenile females. Blue tits occur in the same areas as wood tiger moths and birds may have had previous experience with them, although we cannot ascertain this. The birds were typically tested within a day after they were captured. The birds were weighed again before being released. If the captured birds had not been ringed, they were ringed prior to being released.

The experimental assays were conducted in a standard plywood box (50 x 65 x 45 cm) with a perch where the birds had free access to water. One of its walls consisted of a translucent polyethylene plate with an attached sheet of brown paper to create a visual barrier between the observer and the bird and thus reduce the observer-caused stress experienced by the birds. The paper sheet had a small hole through which the bird behavior was observed. The assays were conducted in a dark room with as little noise as possible to reduce bird stress. Birds were food-deprived for 1 h prior to being tested to ensure they were motivated to hunt and were accustomed to the experimental arena.

### Experiment 1 (with artificial prey): birds’ response to find visual stimuli when viewed under achromatic and chromatic background contexts in a fixed light environment

To test how predators utilize chromatic and luminance information in their prey search, we conducted behavioral assays with wild birds. Fifteen blue tits (*C. caeruleus*) were exposed to two simulated visual contexts: achromatic light-grey (i.e., luminance-rich) background and colorful saturated-yellow (chroma-rich) background. The rationale for using the yellow background is that it allows a more mechanistic comparison of luminance and chromatic cues that are similar to polymorphic wood tiger moth color morphs (see Experiment 2). Both backgrounds were printed on a paper sheet (Xerox A3 80g/m^2^) and created with Swift Publisher 3 software as follows. Achromatic grey background: HSB—Hue 62 degrees, Saturation 1 %, Brightness 90 %; CMYK—C 0, M 0, Y 0, K 8; RGB—R 229, G 229, B 227; HEX#E5E5E3. Chromatic yellow background: HSB—Hue 57 degrees, Saturation 96 %, Brightness 100 %; CMYK—C 0, M 0, Y 100, K 0; RGB—R 255 G 241, B 11; HEX#FF10B.

**Figure 1:**
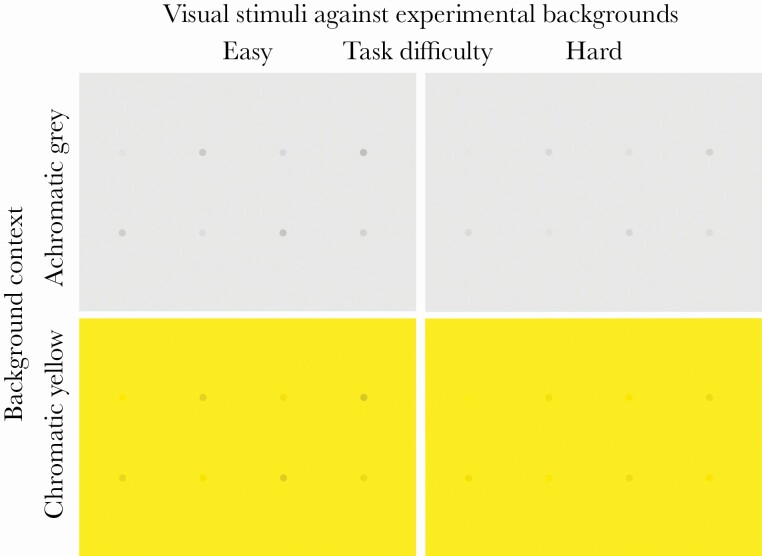
The study was designed to manipulate visual stimuli against chromatic and achromatic background contexts. Blue tits response to find the artificial stimulus was measured in seconds and recorded in four treatments: easy achromatic grey, hard achromatic grey, easy chromatic yellow and hard chromatic yellow. In total, 15 birds were used resulting 60 test plates (four per bird). Each test plate had eight stimuli in randomized order organized into 2 by 4 grid as exemplified in the figure.

A design with two broad difficulty levels (easy, hard) was chosen to make the task easy enough for birds to be willing to co-operate and complete the task while keeping it challenging enough. The difficulty of the task was manipulated by thresholding stimulus with linear increments in CMYK space (Table 1). Each test plate had eight stimuli in randomized order organized into a 2 by 4 grid (Figure 1), which were presented to the bird simultaneously. The distance between two stimuli within the test grid was 10 cm. The “prey” items were stimuli seen against background as differently shaded dots (9 mm in diameter) and presented against either a yellow or a grey background (Supplementary Figure S1, Table 1). The stimuli were directly printed on the background paper as described above, so that shadows would not reveal the location of the stimulus. In the easy task, we manipulated the stimuli by linearly increasing their contrast against the background by 2 percent (in the CMYK space) for a total of eight steps whereas, in the hard task, we used 1 percent increments (Table 1). Thus, easy task stimuli were increasingly more contrasting from the background than the hard task stimuli. The A3 test sheet was placed over a polyurethane plate with small wells carved for placing rewards (i.e., peeled sunflower seeds).

**Table 1:** Manipulating artificial visual stimuli in CMYK space and its relationship to avian vision modelled contrast values (ΔL, ΔS) in two backgrounds designed to test the use of context-dependent chromatic and luminance information using blue tits as a model species. Note that quantum catches are relative; the distances may not be meaningful in absolute terms, as they mirror relative changes in dL and dS values. The absolute stimulus contrast steps describe one percent change in the manipulated CMYK channel.

Yellow—Chromatic background				Grey—Achromatic background			
Stimulus	CMYK	dS	dL	Stimulus	CMYK	dS	dL
Y1	0,0,100,1	0.21	0.04	G1	0,0,0,9	0.22	0.15
Y2	0,0,100,2	0.65	0.22	G2	0,0,0,10	0.01	0.63
Y3	0,0,100,3	1.15	0.57	G3	0,0,0,11	0.27	1.34
Y4	0,0,100,4	1.26	1.74	G4	0,0,0,12	0.26	1.89
Y5	0,0,100,5	1.49	1.58	G5	0,0,0,13	0.72	2.64
Y6	0,0,100,6	2.18	1.36	G6	0,0,0,14	0.54	2.47
Y7	0,0,100,7	3.09	1.78	G7	0,0,100,15	0.92	3.46
Y8	0,0,100,8	2.43	1.34	G8	0,0,100,16	0.62	2.48
Y10	0,0,100,10	2.99	0.77	G10	0,0,100,18	1.03	2.74
Y12	0,0,100,12	3.14	1.65	G12	0,0,100,20	1.00	3.17
Y14	0,0,100,14	3.62	1.55	G14	0,0,100,22	1.67	5.30
Y16	0,0,100,16	2.39	1.72	G16	0,0,100,24	1.23	4.24

Prior to the experiment, the birds were trained to hunt and open up different colored stimuli than those used in the experiment. In the training phase green on brown stimuli were used, whereas in the experimental phase grey shading on either yellow or grey backgrounds were used. Birds were trained to 1) retrieve food (a peeled sunflower seed) placed on the top of a training stimulus, 2) fetch the food from a hole in the training background sheet, 3) search food underneath the training background (i.e., food placed inside the wells) with only a small hole in said background to aid the search, 4) search the food only on a visual basis using the colored stimulus as a cue of the reward placed underneath the sheet. When the bird had learned to pierce through the background paper with visual stimuli to gain the reward (i.e., as in stage 4 above), birds were assigned to experimental backgrounds where they had to repeat this behavior.

Each bird was tested four times: for easy chromatic, hard chromatic, easy achromatic, and hard achromatic tasks presented in random order. No visual barrier was used inside the experimental arena to allow the bird to evaluate the entire visual scene. Each bird conducted all four treatments (as above) in a randomized order and leaving 15 min for recovery between the trials. During the experiment, we recorded the time to find the prey in seconds and if the bird found the item successfully. We recorded the following time variables: active search time (i.e., the time bird was actively “hunting” for visual stimuli on experimental background), split time (i.e., the time it takes from bird to move on to find next prey), and total time (i.e., overall duration in seconds). However, we specifically used only active search time in the analysis because this proved to be the most accurate time variable to measure bird behavior in visual search tasks. The active search time birds were allowed to hunt the prey from a test plate was restricted to 15 min (i.e., 900 s), which corresponds to less than 2 min of processing time per target. In total, 15 birds were used (resulting 60 stimuli plates shown). The light environment was fixed to bright light conditions in this first experiment (see below for further irradiance profiles).

### Prerequisite for experiment 2: measuring and creating natural irradiance profiles

The light environments we used in our second experiment were inspired by the natural forest light environments described by Endler (1993). To achieve this, we measured the irradiance profile of forest shade, small gaps, woodland shade, and large gaps from Boreal forests. Briefly, forest shade represent shaded areas under a canopy. Small gaps are areas located under a canopy that receive direct sunlight through small canopy openings. Both woodland shade and large gaps are not under canopies: the former is shaded by forest edges whilst the latter is under direct sunlight (Endler 1993).

We quantified the light conditions through irradiance measurements, which we repeated at least five times for each light environment within each area across 15 sites. The forested sites were located in Jyväskylä, Central Finland, approximately 10 km southwest from city center, and were spread at least 1 km from each other. The measurements were taken using a Maya 2000-PRO spectrometer model MAYP11351, produced by Ocean Optics (Dunedin, FL), by averaging four scans, using automatic integration times, and by enabling the “electric dark correction” option. These measurements were taken at roughly 1 meter from the ground during clear sky conditions. The fiber cables calibrated with spectrometer were Avantes FC-UV100-2 (0410001) and accompanied with CC-3-UV-S Spectralon optical diffuser cosine corrector (range: 200–2500 nm, optical diameter: 6.35 mm, diffuser diameter 3900 mm, field of view 180 degrees) were used. Additionally, the measurements were taken during intermediate daylight hours in July (the measurements were taken in between 10:00 hrs and 14:00 hrs range), avoiding dusk and dawn as these are known to have different light conditions when compared to regular daylight times (Endler 1993).

**Figure 2:**
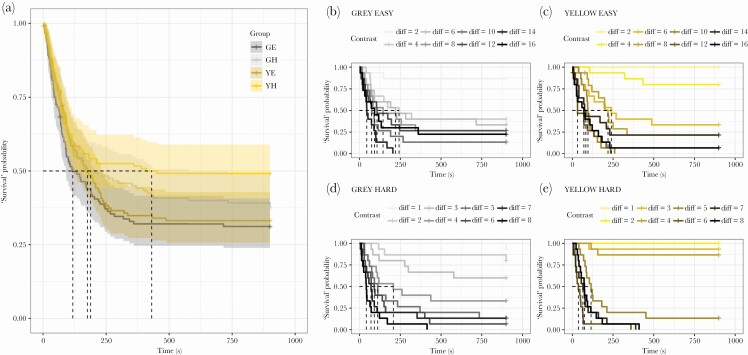
Search time of blue tits to find the test stimuli. The study was designed to manipulate visual stimuli against chromatic and achromatic backgrounds. Blue tits efficiency to find the artificial stimuli (“target survival”) was measured in seconds and recorded in four treatments (A): easy achromatic grey (GE), hard achromatic grey (GH), easy chromatic yellow (YE) and hard chromatic yellow (YH). The group specific Kaplan-Meier plots are plotted separately for GE (B), YE (C), GH (D) and YH (E), which indicate the targets’ contrast difference (“diff”) and their “survival” (i.e., detection) probabilities. The contrast difference to background refers to 1% (hard task) or 2% (easy task) absolute change in the manipulated CMYK channel. In total, 15 birds were used resulting 60 test plates (four per bird) with artificial target stimuli shown for them.

The irradiance profiles indicated differences in boreal forests’ light conditions: the light environments that we recorded showed differences in their irradiance profiles, intensity, or both (Figure 2). Forest shade has the lowest light intensity and high irradiance wavelength values between approximately 450 nm and 570 nm. Large gaps have the highest light intensities with a profile that has high irradiance values for wavelengths of 450 nm onwards. Small gaps were similar to large gaps but the irradiance intensity found on the former was smaller than the irradiance found on the latter. Woodland shade had high irradiance intensities with wavelength values between 450 nm and 550 nm. The most contrasting light environments were therefore large gaps (high light intensity) and forest shade (low light intensity). Thus, we may assume that in forest shade conditions short wavelengths may be more important in biasing predator decisions than in bright light conditions where chromatic cues should be more effective within receivers’ sensory capabilities.

We used the irradiance profiles of forest shade and large gaps as reference for recreating the forested and open area light environments used during the behavioral experiment (see below). These two were chosen as they represented two contrasting light environments, and their irradiance profiles are markedly different in intensity. Thus, we may expect the influence of light environment to escalate differences in predator decision-making behavior towards color polymorphic (white and yellow) defended prey.

### Experiment 2 (with real prey): influence of altered light environment to survival of polymorphic prey

We used simultaneous choice tests with additional 49 blue tits (i.e., different individuals than in the previous experiment) to assess how different light conditions might affect polymorphic wood tiger moth yellow (i.e., chrome-rich) and white (i.e., luminance-rich) warning signal efficacy in deterring predators. Specifically, we tested which moth coloration blue tits preferred to attack and/or avoided more under light conditions naturally found in open and forested areas. Although sequential tests may give a more independent estimate of the attack preference over time, as our aim here was to offer choice to see to which moth blue tits would go first, we opted for simultaneous choice tests. By conducting these assays, we tested interactions among coloration, light conditions, and signal effectiveness. We did not use trials with birds that did not attack the moths in our analysis.

**Figure 3:**
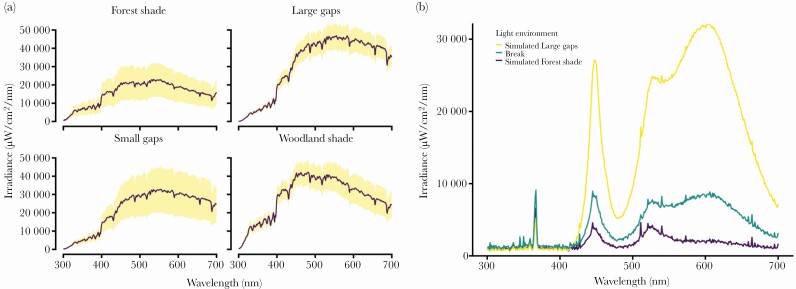
Irradiance profile of Forest shade, Large gaps, Small gaps and Woodland shade measured from the Boreal forest zone in Finland (A). The purple line represents the mean value of the irradiance measurements taken within these different environments. The yellow shaded area shows the standard deviation of the irradiance measurements. Irradiance profile of the light environments used in the behavioral assays was designed to mimic naturally occurring conditions (B). In the experiment, we manipulated irradiance to produce two contrasting light environments simulating open (i.e., high-intensity) and forested (i.e., low-intensity) conditions.

To simulate different forest light conditions, the experimental arena was equipped with two Philips Hue white and color ambiance E26 light lights (operating in between 400–700 nm range) and a ReptiGlo UVB lamp in tandem with a Baader U filter (providing 320–380 nm range). These Philips light LED lights allowed for light wavelength customization and thus were used in combination with the UV producing lamp to create light conditions simulating the irradiance profile of open (i.e., high light intensity) and forested (i.e., low light intensity) conditions. We also used an intermediary profile to provide a neutral “break” (as depicted in Figure 3B) light environment for the birds to rest in during the experiment and to make the transition between the light environments smoother (Figure 3). We called the stage with the intermediary profile “break.”

Pre-training was conducted in the aviaries. The birds’ food was removed, and they were offered a peeled sunflower seed on a green cardboard coaster with a diameter of 9 cm. This was done in order to habituate the birds to forage on the green coaster. These coasters were also used in the remaining steps of this experiment. Once the first seed was eaten, another seed was offered, and this process was repeated until the birds had eaten five seeds. The experimental protocol consisted of four stages: familiarization, experimental stage I, break, and experimental stage II.

During familiarization, the birds were kept under the intermediary “break” light profile and were food-deprived for 30 min. This was done in order to habituate birds to the experimental box and to increase their motivation to hunt in a controlled manner. The birds were then offered a mealworm (a *Tenebrio molitor* larva) weighing 8–12 milligrams on a green coaster to test their motivation: if the bird being tested ate the offered mealworm within 3 min, we proceeded to next stage, if not, we waited 5 min and repeated this stage starting from offering the mealworm.

During experimental stage I, we switched the light conditions to either simulated forested or open area and waited 5 min for the bird to habituate to the new light conditions. The initial choice of which light environment to use during this stage was random and the following trials were done alternating the order in which the light environments were tested. After these 5 min, we offered the birds a yellow and a white moth (i.e., being a simultaneous choice test) fixed on a green coaster using adhesive tape. The moths were dead, frozen, and thawed about an hour before the experiment started. The moths (originating from the laboratory stock) were placed on opposite sides of the circular coaster’s diameter. Their wings were slightly opened so that they would represent the natural resting position of the moths, but still enable visual cues of the hind wings to be assessed. We then observed whether the bird attacked the yellow or the white moth first. Only definitive attacks were included in the analysis: the bird had to pick up the moth for its action to be considered an attack. If the bird being tested attacked a moth within 3 min, we proceeded to the next stage, if not, we removed the moths, waited 5 min, and offered the moths again. Noteworthily, birds generally attacked the moths in both trials and as we recorded the first attack towards the moths, we were excluding further assessing the role of secondary defenses.

During the break stage, we exposed the bird to the intermediary profile and waited 10 min. This was done to give the birds a rest and to make the light environment transition smoother. Then, we tested the birds’ motivation again: another mealworm was offered and if the bird ate it within 3 min we proceeded to the next stage.

For experimental stage II, we set the light conditions to the light environment that was not used during experimental stage I. We then waited 5 min and offered the two different-colored moths fixed on a green coaster. We switched the moth position on the coaster between the experimental stages I and II to reduce the chances of the birds’ choice being affected by this.

### Avian vision modeling

To link visual perception to behavior in a more mechanistic manner, we implemented avian vision modeling. Briefly, these models estimate how animals perceive visual stimuli and thus, enable us to tease apart the effects chromatic and luminance contrasts against the background. The obtained contrasts were combined with the data from the experiment to investigate how they might influence bird decision-making in contrasting light environments. The required reflectance measurements were performed using a Maya 2000-pro spectrometer model MAYP11351, produced by Ocean Optics (Dunedin, FL). We used blue tit vision system: the four categories of single cones regarding their maximum wavelength sensitivities (λ _max_) and their relative frequencies (inside brackets) were UVS λ _max_ 372 nm (0.37), SWS λ _max_ 413 nm (0.71), MWS λ _max_ 508 nm (0.99) and LWS λ _max_ 573 nm (1.00) after (Hart et al. 2000). The achromatic sensitivity was simulated by using double-cones, which appear to be sensitive to long-wavelength light, but are assumed to serve achromatic tasks, such as luminance and motion sensing, instead of chromatic vision (Osorio and Vorobyev 2005).

The vision models were used to calculate the raw photon catch values (quantum catches or Qi) for each different photoreceptor modeled, which then yielded the chromatic and luminance contrasts between wing coloration and the background. These contrasts were calculated in units of ΔS (Weber fraction 0.03 was used) and ΔL (Weber fraction 0.08 was used) for chromatic and luminance contrast (Silvasti et al. 2021), respectively. The model yield values as just noticeable differences (JNDs), which are defined as the minimum change in stimulus intensity that yields a noticeable variation in sensory experience whereby values < 1 are considered non-detectable (Vorobyev and Osorio 1998). To calculate the reported contrast values in experiment 1, we took 3 reflectance measurements of each visual stimulus and compared these to the respective background. In experiment 2, we took measurements of 11 moths of each color morph and modeled their hindwing contrast against the green experimental background and between the moths. Models were built using an R package “pavo” (Maia et al. 2013, 2019).

### Statistical analysis

#### Experiment 1 (with artificial prey)

To test active search time to find the prey, we used a mixed-effect Cox model (coxme). We set “survival” (0,1) as response with time to find the prey (in seconds), whereas background (grey achromatic, yellow chromatic), task difficulty (easy, hard), and stimulus contrast (difference to background) were set as its fixed factors together with their interactions. Bird ID and the order in which the plates were offered were set as random factors. To dismantle the relationship across background type and stimulus contrast, we split the data according to achromatic versus chromatic background, and re-run the analysis for both background types separately using avian vision modeled contrast values. For this, we used ΔL in achromatic background and ΔS in chromatic background—although the obtained contrast values correlate with linear increments of contrast steps, which are independent of receiver vision system.

#### Experiment 2 (with real prey)

To test if birds’ preference regarding attacking one of the two differently colored moths depended on the light environment being simulated, we analyzed the data from the behavioral assays using generalized linear mixed models (glmm) with a binomial error distribution. The initial model had the first attacked moth color morph as its response variable and the interaction between light environment and light environment testing order, their main, separate effects, bird sex, and bird age as fixed factors. The bird identity was a random factor to account for the fact that each bird was tested in two different light environments.

#### Data accessibility

The supporting data is archived in an appropriate public repository (Nokelainen et al. 2021); DOI: 10.17011/jyx/dataset/77737. All tests are two-tailed tests and full models are reported. Analyses were conducted using R-studio -software, version 1.1.447 and R, version 3.5.0 (RStudio Team, 2016; R Core Team, 2018).

## RESULTS

### Experiment 1 (with artificial prey): birds’ response to find visual stimuli when viewed under achromatic and chromatic background contexts in a fixed light environment

The active search time (i.e., how quick the birds were able to complete the task to find the target) was used to assess birds’ response to find visual stimuli under luminance and chromatic contexts (Figure 2A, Table 2). The active search time was influenced by a significant three-way interaction (_background*difficulty*contrast_: Z = –1.25, *p* < 0.001). To dismantle this interaction, we split the data according to achromatic versus chromatic contexts, and re-ran analysis for both background types separately (Table 3). Birds found the stimuli faster when the task was easy irrespective of the background type (Figure 2B–C), but when the task was hard (Figure 2D–E), birds found the stimuli faster against chromatic background, but only to a certain point. More specifically, blue tits took advantage of chromatic cues leading to faster prey detection in the chromatic yellow background in comparison to when only luminance cues were present in the achromatic light-grey background, but the latter remained detectable in lower reflective intensities (i.e., birds detected smaller contrast differences). This was evident when the task was difficult to achieve, but there was no effect when the task was easy (Figure 2, Supplementary Figures S1–S2, Tables 1–3).

**Table 2:** The descriptive information of active search time (i.e., how quick the birds were able to complete the task to find the prey) of blue tits to find artificial visual stimuli broken down by background treatments. The mean value to search one stimulus at time can be obtained by dividing the presented values by 8 as each test plate had eight stimuli simultaneously presented.

Background	Mean	SD	Median	Range	SE
Grey easy	349.21	380.06	116.50	895	34.69
Grey hard	428.55	395.40	188.50	894	36.09
Yellow easy	370.86	377.38	173.00	897	34.45
Yellow hard	349.82	409.84	431.00	894	37.41

**Table 3:** The effects of visual contrast on predator’s search efficiency. To test active search time to find the stimuli, we used a mixed-effect Cox model. The table is split in visual background contexts (achromatic grey, chromatic yellow). The model terms refer to task difficulty (easy, hard), and contrast of the stimuli (difference to background). The bird ID and the order in which trays were presented are set as random factors in both models. In chromatic context birds found the stimuli faster with increasing contrast of the stimuli in comparison to achromatic cues only, whereas smaller contrast differences *per se* were found in achromatic background.

Model term	coef	exp(coef)	se(coef)	Z	*P*
Achromatic grey					
Difficulty	1.24	3.47	0.43	2.86	<0.001
Contrast (dL)	1.78	5.98	0.27	6.43	<0.001
Contrast (dL) * Difficulty	–0.67	0.50	0.15	–4.51	<0.001
Chromatic yellow					
Difficulty	1.09	3.01	0.49	2.24	<0.001
Contrast (dS)	1.94	7.01	0.31	6.33	<0.001
Contrast (dS) * Difficulty	–0.56	0.57	0.18	–3.05	<0.001

### Experiment 2 (with real prey): influence of altered light environment to survival of polymorphic prey

**Figure 4:**
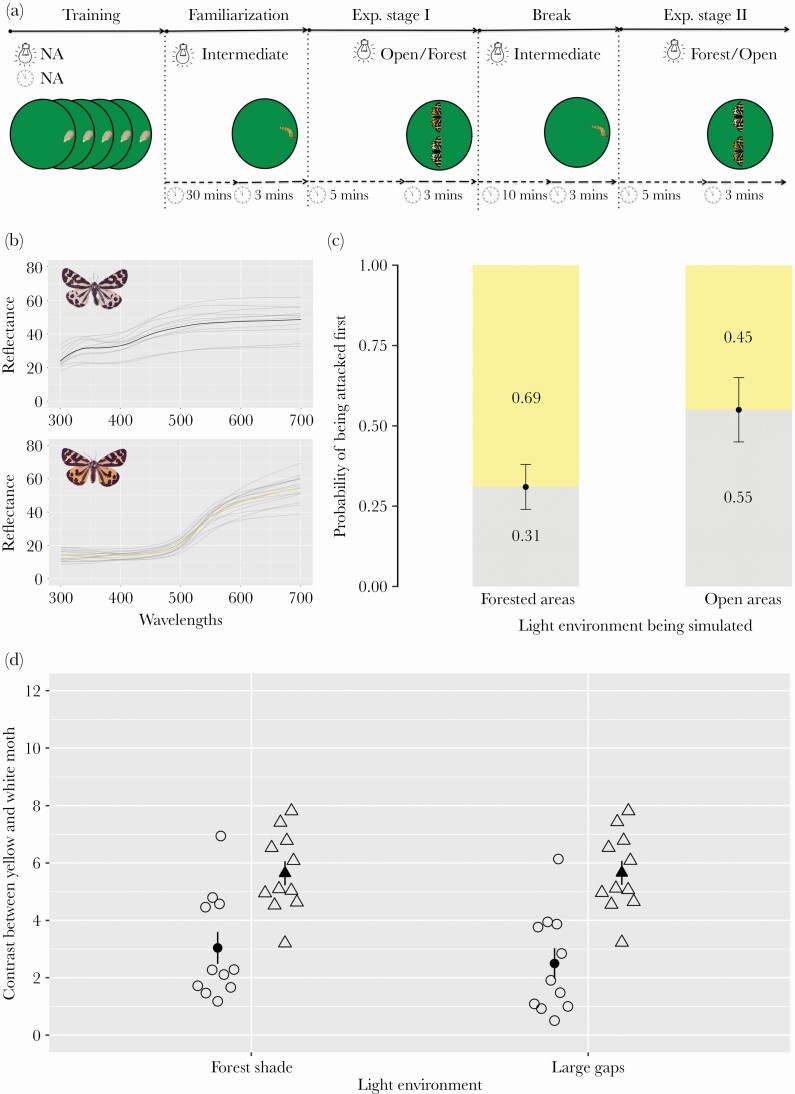
Simultaneous choice tests using blue tits (*n* = 49) as predators and two different morphs of wood tiger moth as prey. A) Prey were offered in two different light environments: “forest” (low light) and “open” (bright light). The light bulbs denote the light conditions used during a specific stage and the clocks the time allocated to each stage. “Break” stands for intermediate light profile in-between the two experimental light environments. B) The white morph has higher reflectance in short wavelengths (and luminance) in comparison to the yellow morph. C) Light conditions influenced predators’ choice between yellow and white *A. plantaginis* males. The *x* axis represents simulated forest and open light conditions. The *y* axis represents the bird attack probability derived from the GLMM model. D) Avian vision modeled contrast comparison between yellow and white morph: circles show luminance, triangles show the chromatic contrast and the bars denote plus/minus 1 SE.

We manipulated the experimental light environment to test (Figure 4A) whether the color morph (Figure 4B) blue tits chose to attack first depended on the light environment. Under simulated open areas white moths had a probability of 0.55 of being attacked first while yellow moths had a probability of 0.45 (*Z* value = 2.42, d.f. = 1, *p* = 0.015, Table 4, Figure 4). Specifically, 15 white moths and 34 yellow moths (total 46 moths—in three cases we could not determine which moth was attacked first) were attacked first under simulated forest light condition (i.e., low light intensity), whereas 24 white moths and 22 yellow moths (in total 49 moths) were attacked first under light condition simulating open areas (i.e., bright light intensity). Under simulated forest light, white moths were less likely to be attacked compared to yellow moths (*Z* value = –2.64, d.f. = 1, *p* = 0.008, Table 4, Figure 4C). Thus, birds’ choice may change based on the visual cues depending on the context.

**Table 4:** The GLMM predicted effect of different light environments to predator preference when choosing between chemically defended yellow and white wood tiger moth males. The estimates refer to the probability of birds attacking white males under the two light environments. The probability of birds attacking yellow males is complementary to the probability of birds attacking white males.

Model term	Estimate	Standard error	*Z*	*P*
Intercept [low light env]	–0.81	0.31	–2.64	0.008
High light environment	1.02	0.42	2.42	0.015

Elusively, the avian vision model obtained luminance and chromatic contrast values (ΔL and ΔS) showed that the light environment did not significantly change the perceived contrast (Figure 4D, also see discussion). White moths had higher luminance and yellow moths had higher chromatic contrast in both light environments (Figure 4D). Noteworthily, albeit statistically non-significant result, when comparing morphs directly together in bright light conditions chromatic contrast was predicted to be perceptible for both morphs, whereas luminance contrast was predicted to be just noticeable in certain cases (i.e., ΔL close to 1). In turn, both luminance and chromatic contrasts were always predicted as perceptible (i.e., ΔL and ΔS > 1) in low light conditions. In any case, the vision model results were not sensitive enough to detect noticeable changes in perceived contrasts with respect to experimental irradiance despite our results show that light environments may change predator decision making.

## DISCUSSION

We demonstrate that birds show context-dependent predatory behavior and that the light environment influences predator decisions regarding whether to attack color polymorphic prey. Using artificial stimuli in controlled conditions, we first showed that bird predators utilized chromatic and luminance cues differently depending on task difficulty. As a follow-up, we created two light environments in experimental conditions, which were sufficiently different to influence attack probability of real prey. However, our vision modeling results did not support different luminance and chromatic contrasts in between the two light environments experimented, which we discuss further.

A geographic mosaic theory of coevolution (Thompson 1999) can predict that heterogeneous predator selection may facilitate multiple prey appearances (Rönkä et al. 2020). With this respect, variable light conditions have been identified as a potential source of heterogeneous selection that could result in the emergence of such phenotype variation (Rojas et al. 2014; Tate et al. 2016; Passarotto et al. 2018; Kranz et al. 2018). We found that predation risk on the different-colored *A. plantaginis* morphs was dependent on the light conditions that prey morphs and their predators were experiencing. Blue tits were more likely to attack yellow *A. plantaginis* males under simulated forest light conditions, but when the light conditions of open areas were being simulated, white males were more likely to be preyed upon. Thus, if forests provide a net benefit in survival to white individuals and open areas provide a similar advantage to yellow individuals, both morphs may survive since these environments are found side by side in semi-open woodlands. Such mosaic selection could contribute to the maintenance of color polymorphism if the resulting differences in survival are enough to make the light environments act as sources or sinks for the color morphs (Thompson, 1999).

Birds categorize chromatic cues as humans do (Caves et al. 2018; Kelber 2019). In agreement, our results suggest that targets presented in a chromatic context were found faster, but sharply to the limit where they remained perceptible after which search times quickly deteriorated to become ineffective at low contrast intensities (i.e., showing a threshold change in detectability). As luminance perception often shows a logarithmic response to changing light intensity (Troscianko et al. 2017; van den Berg et al. 2020), this could explain a linear response of search times under achromatic context because searching for prey still takes a certain time (i.e., higher detectability may not lead to faster foraging). The detectability of luminance and chromatic contrasts could be coupled with changing light environments. An interesting outcome of this may be that warning signals may not need to be “honest” in a manner that the most defended organisms “signal the loudest” (Maan and Cummings 2012; Arenas et al. 2014; Rojas et al. 2018). Instead, in variable light environments, signals being categorized as unprofitable may be enough either due to generalization for the common signal or simply by possessing conspicuous coloration (Ham et al. 2006; Rönkä et al. 2018b).

The warning signal of *A. plantaginis* male morphs differ in their chromatic and luminance contrasts according to the blue tit vision models we ran (Nokelainen et al. 2012). Differences in luminance might be more readily detectable by predators than chromatic contrast differences under low light conditions, since discerning colors can be constrained by scarce luminosity (Penteriani and Delgado 2017). However, our vision modeling results did not support that light environment skews the detectability of the two morphs (Henze et al. 2018). Instead, the luminance and chromatic contrasts were relatively constant in the two light environments. A further examination of the irradiance component (i.e., blue sky, D65, forest shade, ideal; the latter referring to homogeneous illuminance of 1 across wavelengths) showed that the blue tit vision model predicted similarly high contrast values of yellow and white tiger moths irrespective of the light environments (Maia et al. 2013, 2019). Either this means that the vision model is too sensitive (or not sensitive enough) to account for light being sufficient to detect the perceived contrast differences or, alternatively, that birds decide, which morph to attack based on higher-level cognitive processing. As higher-level cognitive processing takes place later in the decision-making process to attack the prey than what the model accounts for (i.e., reflectance, irradiance, retinal anatomy), it may be that vision modeling does not completely cover predator decision-making process. To verify vision model performance, detailed behavioral assays of color discrimination should take place. Nevertheless, the birds did change their attack preference. This suggests context-dependency; predators may base their attack decision on different environmentally-mediated cues (Hansen et al. 2010), luminance in dark, and color in bright light, albeit their relative contrasts would be predicted to remain perceptible (van den Berg et al. 2020).

Taken together, our results suggest that changing light environment and following alternated predator behavior can ultimately lead to mosaic-like selection dynamics across the landscape (Thompson 1999). Receiver psychology is important to understand how cognition biases decision-making (Guilford and Dawkins 1991; Rowe 2013; Endler and Mappes 2017). The key conjecture is how predators assess their prey: in the case of predators associating defended prey with luminance cues, these cues may work as warning signals in the respective light environment. Variation in coloration is likely to be maintained through the joint effect of several mechanisms, making it hard to detect their individual effects. There are reports describing the effects of heterozygote advantage, variation in predators at different levels, socially transmitted avoidance, signaling trade-offs, among others as mechanisms facilitating variation in coloration and/or color polymorphism (Exnerová et al. 2010, 2015; Nokelainen et al. 2014; Willmott et al. 2017; Thorogood et al. 2018). There is no reason to believe mechanisms that influence success of visual cues act in isolation from each other. Their subtle, intertwined action can make it hard to detect the magnitude of those effects in a natural setting. Still, this does not mean that these mechanisms’ individual effects should be neglected; they do affect fitness regardless of how difficult it is to measure such effects.

## Supplementary Material

arab111_suppl_Supplementary_Information

## Data Availability

Analyses reported in this article can be reproduced using the data provided by Nokelainen et al (2021).
