# Supplementary material for: Context-dependent coloration of prey and predator decision making in contrasting light environments
Source: Behav Ecol. 2021 Oct 18;33(1):77–86. doi: 10.1093/beheco/arab111 (PMC8857938; doi:10.1093/beheco/arab111)

Supplementary material

Title: Context-dependent coloration of prey and predator decision making in contrasting light environments

Authors: Ossi Nokelainen[1*], Francisko de Moraes Rezende[1], Janne K. Valkonen[1], Johanna Mappes[1]

Addresses: [1]Department of Biological and Environmental Science, University of Jyväskylä, P.O. Box 35, 40014 University of Jyväskylä, Finland

E-mail addresses: ossi.nokelainen@jyu.fi, francisko.rezende@gmail.com, janne.valkonen@jyu.fi, johanna.r.mappes@jyu.fi, [*] Authors for correspondence (ossi.nokelainen@jyu.fi)

Running headline: Context dependent predation in light environments

Data accessibility statement: The supporting data is archived in an appropriate public repository (DOI: 10.17011/jyx/dataset/77737).

Contents: Page

Supplementary figures

Figure S1 2

Figure S2 3

Figure S1: Experimental background used in this experiment. A) Examples of the visual stimuli against experimental backgrounds. Here, unlike in the experiment, the visual stimuli are encircled for easy visualisation of the grid arrangement. B) In experimental background, the shading of visual stimuli is subtle, illustrating the task difficulty depending on the background context. Blue tits response to find the artificial stimulus was measured in seconds and recorded in four treatments: easy achromatic grey, hard achromatic grey, easy chromatic yellow and hard chromatic yellow.

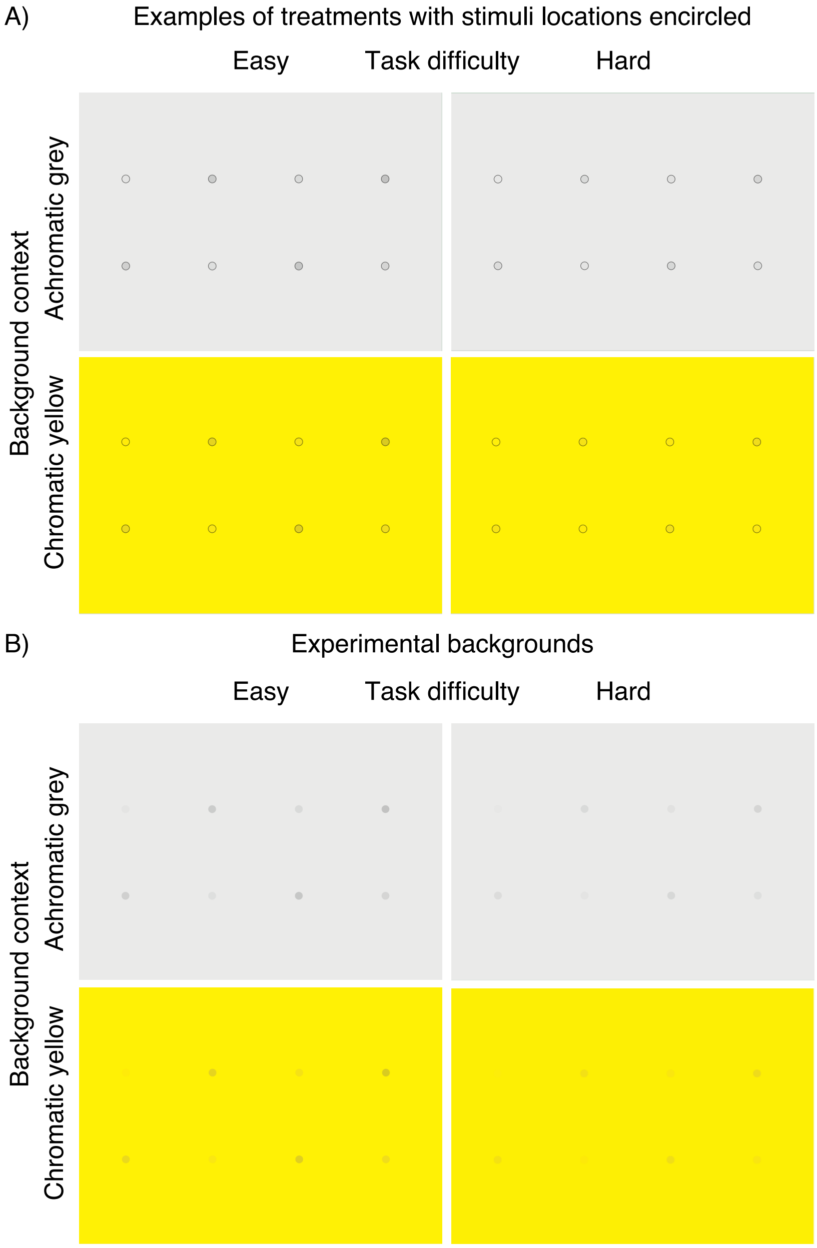

Figure S2: Blue tits' search efficiency to find the test stimuli. The study was designed to manipulate visual stimuli in chromatic and achromatic background contexts. The inset picture (A) shows the visual search task appointed to blue tits. Stimuli were designed to be very similar to the background (Fig. S1), as indicated by Maxwell's colour triangle (B) and avian colour space tetrahedron (C). Blue tits response to find the artificial stimulus was measured in seconds and recorded in four treatments (D): easy chromatic, hard chromatic, easy achromatic and hard achromatic. The difference to background is plotted against avian vision model contrast values, where each step on x-axis represents one percent (hard task) or two percent (easy task) absolute change in the manipulated CMYK channel. In total, 15 birds were used resulting 60 test plates (four per bird) with artificial stimuli shown.

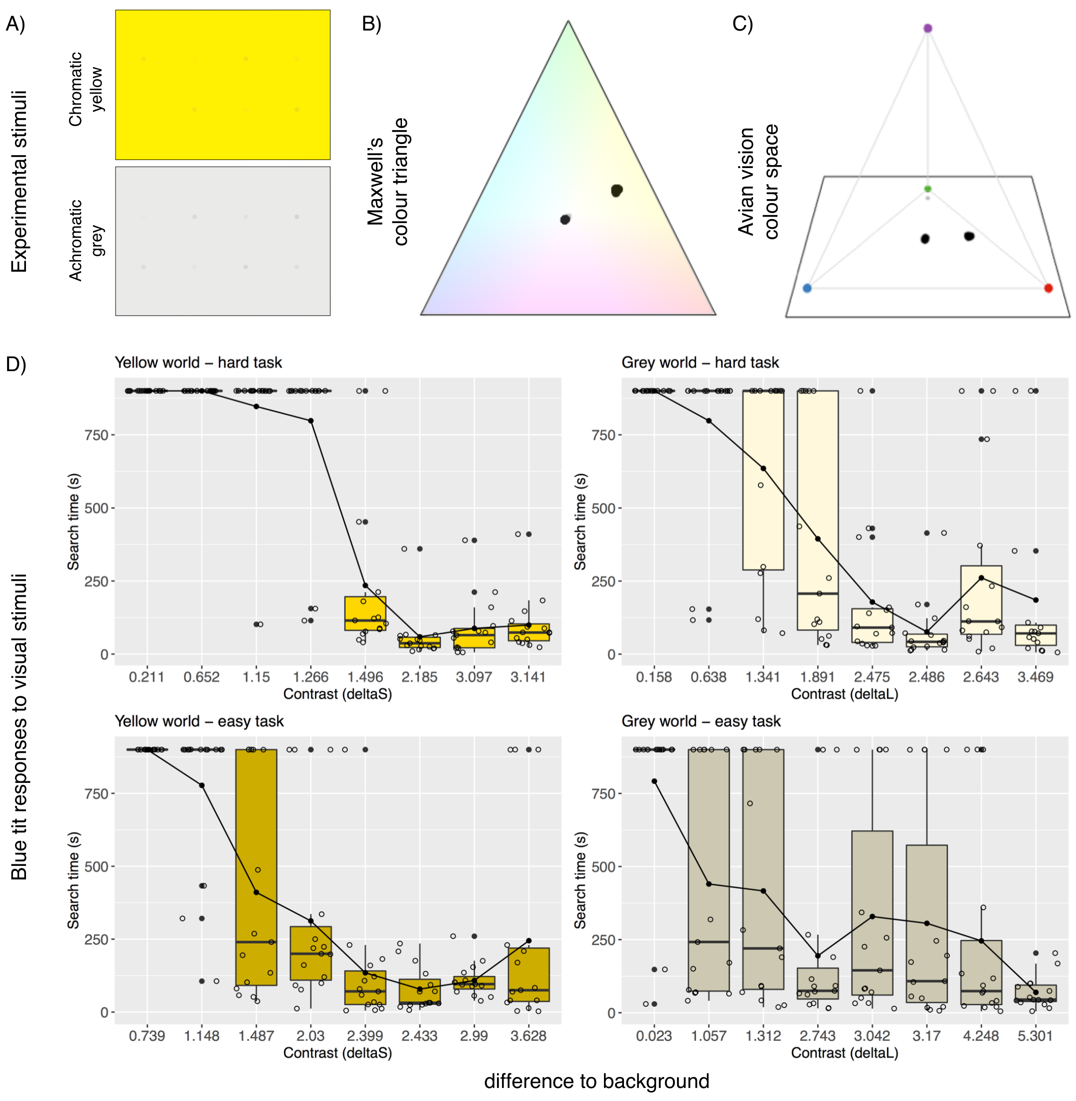

Supplement: arab111_suppl_Supplementary_Information [file arab111_suppl_supplementary_information.pdf]
